# Supplementary material for: Polymorphisms in Pfkelch13 domains before and after the introduction of artemisinin-based combination therapy in Southwest Nigeria
Source: PLoS One. 2025 Mar 31;20(3):e0316479. doi: 10.1371/journal.pone.0316479 (PMC11957316; doi:10.1371/journal.pone.0316479)

Samples: 11987
Bases: 1054
Average spacing: 12

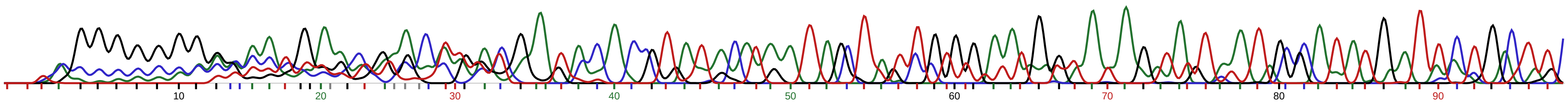

T T T A G G G G G G G G C C A A T G G A - G T A - - - C T T G A C G A A T A C A C G T A T A C T A A T A C T A T T G T G T G A A T G G T A T A G A A T T A A T G C C A T A T G A T T C T G C T T

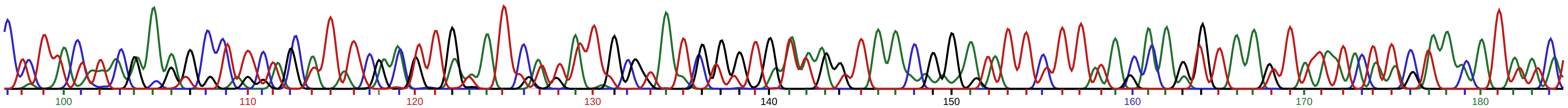

C T T A C T C A A A G C C T C T C T T T C - C T T G A A T C T T A T G C T A T G G G T G A A A G T A A C G G A T T T C T T T A C A A G G T A A T T A T T C T T C A A C A T A A C

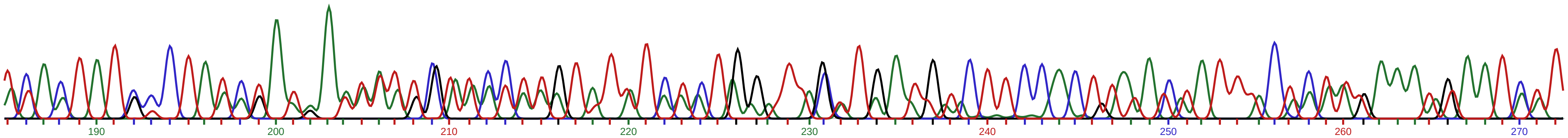

T C A C T A T C C C T A T C T A T A A A T A T T C T T C C T T G T A T A T C T C T G G A T A C T T G A T G T C T T C C A C T T A A C T A T T A C T C T T G A A A T G A A T C T T

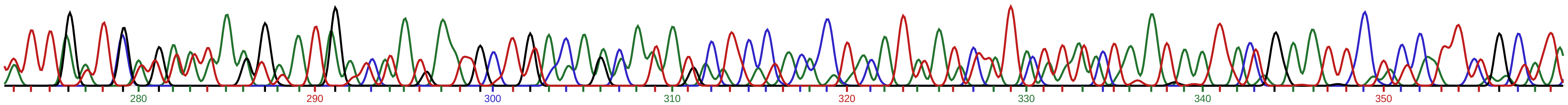

T T T G A T G A G A A T A A G A A T G A C A A T A T G C T G A C A A C A T A T C T C C T C A C T C A T A A T C A T A T T A C T A A T A A T A C G A A T T C T C C T T T G C A T

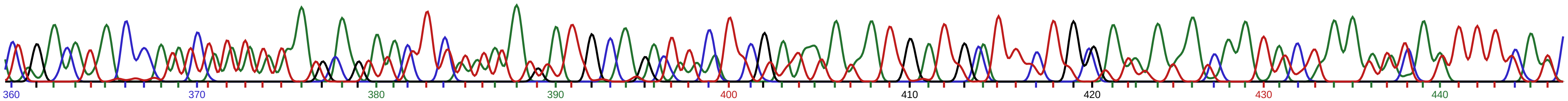

C G A A T A C C A A C T T T T T A G A G A A C T C T T A A T A T G C A G A T T C T C G A T A A T A T G A T G A T T C T G G A T A A T A C A A T A C T A A A T T A A T T T C A T

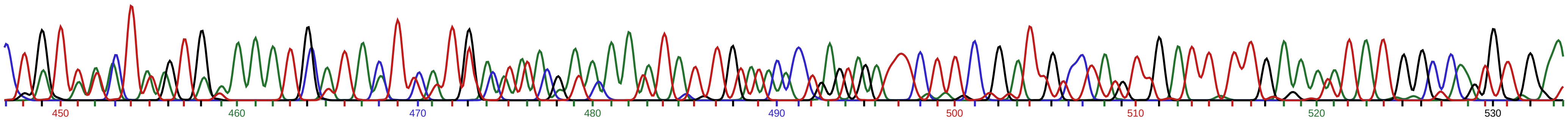

C T G T T A C T T G T G A A A A T G A T A C T C A T G A T A A G A A A A A T A T T G T C C T A A G A T C T T C G A T G C C A G T G A T T T T G A A A A T A T G G T A T G T G A A

Samples: 11987
Bases: 1054
Average spacing: 12

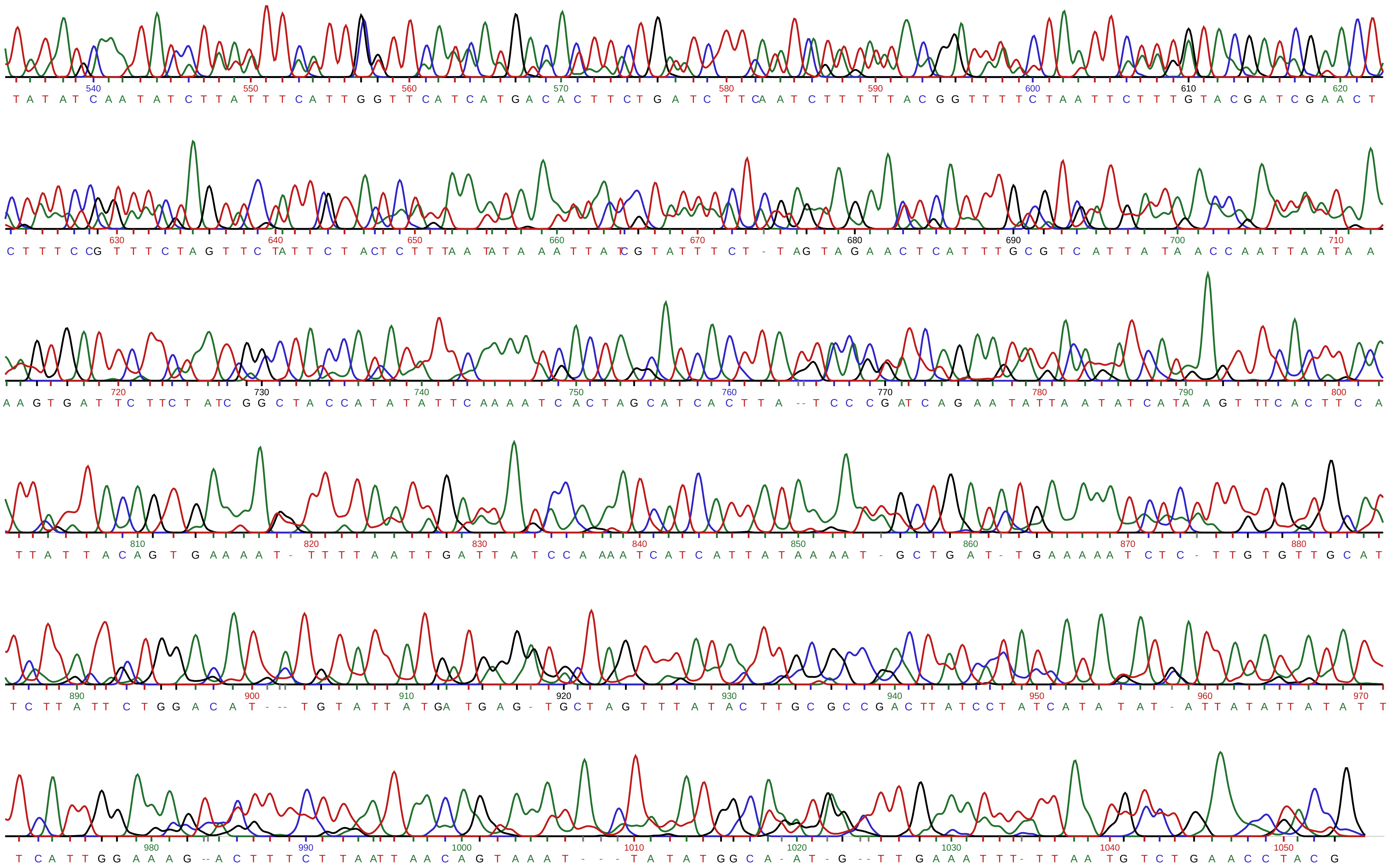

Supplement: Supporting information 2 — (ZIP) [file pone.0316479.s002.zip › 046KN2F_PREMIX_Plate_KELCH2_A03.pdf]
